# APACHE III Outcome Prediction in Patients Admitted to the Intensive Care Unit with Sepsis Associated Acute Lung Injury

**DOI:** 10.1371/journal.pone.0139374

**Published:** 2015-09-30

**Authors:** Zhongheng Zhang, Kun Chen, Lin Chen

**Affiliations:** Department of critical care medicine, Jinhua municipal central hospital, Jinhua hospital of Zhejiang university, Zhejiang, P. R. China; Medical University of South Carolina, UNITED STATES

## Abstract

**Background and objective:**

Acute Physiology and Chronic Health Evaluation (APACHE) III score has been widely used for prediction of clinical outcomes in mixed critically ill patients. However, it has not been validated in patients with sepsis-associated acute lung injury (ALI). The aim of the study was to explore the calibration and predictive value of APACHE III in patients with sepsis-associated ALI.

**Method:**

The study was a secondary analysis of a prospective randomized controlled trial investigating the efficacy of rosuvastatin in sepsis-associated ALI (Statins for Acutely Injured Lungs from Sepsis, SAILS). The study population was sepsis-related ALI patients. The primary outcome of the current study was the same as in the original trial, 60-day in-hospital mortality, defined as death before hospital discharge, censored 60 days after enrollment. Discrimination of APACHE III was assessed by calculating the area under the receiver operating characteristic (ROC) curve (AUC) with its 95% CI. Hosmer-Lemeshow goodness-of-fit statistic was used to assess the calibration of APACHE III. The Brier score was reported to represent the overall performance of APACHE III in predicting outcome.

**Main results:**

A total of 745 patients were included in the study, including 540 survivors and 205 non-survivors. Non-survivors were significantly older than survivors (59.71±16.17 vs 52.00±15.92 years, p<0.001). The primary causes of ALI were also different between survivors and non-survivors (p = 0.017). Survivors were more likely to have the cause of sepsis than non-survivors (21.2% vs. 15.1%). APACHE III score was higher in non-survivors than in survivors (106.72±27.30 vs. 88.42±26.86; p<0.001). Discrimination of APACHE III to predict mortality in ALI patients was moderate with an AUC of 0.68 (95% confidence interval: 0.64–0.73).

**Conclusion:**

this study for the first time validated the discrimination of APACHE III in sepsis associated ALI patients. The result shows that APACHE III score has moderate predictive value for in-hospital mortality among adults with sepsis-associated acute lung injury.

## Introduction

Intensive care unit (ICU) patients are at high risk of death and many risk stratification scores have been developed for outcome prediction. The most widely used scores include Acute Physiology and Chronic Health Evaluation (APACHE) from I to IV, sequential organ failure score (SOFA) and simplified acute physiological score (SAPS). These risk assessment scores are thought to play an important role in evaluating new therapies, triaging patients, improving quality assessment and monitoring resource utilization. However, these scores are mostly developed in unselected ICU patients. For instance, the APACHE III score was derived from 17,440 unselected medical/surgical ICU adult patients [1]. Although the APACHE III score has been well validated in unselected ICU patients [2], it has been found to be less reliable in specific subgroups of critically ill patients. For example, APACHE III predicted moderately between survivors and non-survivors of patients after orthotopic liver transplantation [3], with an area under curve (AUC) of 0.65. Similar results were found with other versions APACHE scores [4, 5], and these scores usually require modification to improve their discriminative power in specific subgroups of ICU patients.

Acute lung injury (ALI) is common in the ICU and is associated with adverse outcomes such as prolonged length of stay in ICU, increased financial cost and the risk of death [6, 7]. Diagnosis and prevention of ALI is an active area of research in critical care medicine. More importantly, the prognostication and triage of ALI patients may help to better utilize medical resources, inform patients and their families, and early use of certain interventions. However, there is currently no prediction score developed specifically for ALI patients. APACHE III is widely used in clinical practice and research for ALI patients, but its validity in sepsis-associated ALI is not systematically investigated. Therefore, the aim of the study was to investigate the discrimination and calibration of APACHE III in predicting mortality in sepsis associated ALI patients.

## Methods

### Data acquisition

The study was a secondary analysis of a prospective randomized controlled trial investigating the efficacy of rosuvastatin in sepsis-associated ALI (NCT00979121)[8]. The study was also known as Statins for Acutely Injured Lungs from Sepsis (SAILS) and performed from 2010 to 2013. The original study was approved by institutional review board at each of the 44 enrolling hospitals. The study stopped after enrollment of 745 of an estimated 1000 patients because of the futility of the intervention. Dataset for this trial was obtained from National heart, lung, blood institute (NHLBI) Biologic Specimen and Data Repository Information Coordinating Center (https://biolincc.nhlbi.nih.gov/studies/sails/?q=rosuvastatin). The secondary analysis was approved by the ethics committee of Jinhua municipal central hospital and informed consent was waived due to retrospective nature of the study. Patient records/information was anonymized and de-identified prior to analysis.

### Study population

Patients were eligible for enrollment if they fulfilled the criteria of sepsis and ALI [8]. Sepsis was defined as documented or suspected infection and two or more of the following criteria for a systemic inflammatory response: 1) a white-cell count of more than 12,000 per cubic millimeter or less than 4000 per cubic millimeter or a differential count with more than 10% band forms; 2) a core body temperature of more than 38 or less than 36 degree centigrade; 3) Heart rate (> 90 beats/min) or receiving medications that slow heart rate or paced rhythm [9]. ALI was defined as acute onset of the illness and all of the three criteria: 1) a ratio of the partial pressure of arterial oxygen (Pao2) to the fraction of inspired oxygen (Fio2) of 300 or less; 2) bilateral infiltrates on chest radiography that were consistent with pulmonary edema, without evidence of left atrial hypertension; and 3) receiving positive-pressure mechanical ventilation through an endotracheal tube [10]. Study coordinators screened participating ICUs daily to identify potential candidates for enrollment.

### Data extraction

Because the original study showed neutral effect of rosuvastatin on mortality outcome, data on the use of rosuvastatin was not considered in our analysis. The following data were extracted: demographics such as gender, age and ethnicity; the type of ICU; the number of quadrants with infiltrates on chest X-ray; suspected or documented infection site; the primary causes of lung injury; and APACHE III score. Variables employed to calculate APACHE III score were obtained 24 hours preceding randomization. Intraoperative values and values associated with death or cardio/respiratory arrest situations were not used.

### Study endpoint

The primary outcome of the current study was the same as in the original trial, 60-day in-hospital mortality, defined as death before hospital discharge, censored 60 days after enrollment.

### Statistical analysis

Descriptive data were summarized as mean (SD) or frequency (percentage) as appropriate. The difference between survivors and non-survivors were compared using student t test for continuous variables and using Pearson’s Chi-square test for discrete variables. If any continuous variables were non-parametric, Mann-Whitney U test was employed.

Discrimination of APACHE III was assessed by calculating the area under the receiver operating characteristic (ROC) curve (AUC) with its 95% CI. Discrimination was classified into categories of excellent, very good, good, moderate, and moderate for respective AUCs of 0.9 to 0.99, 0.8 to 0.89, 0.7 to 0.79, 0.6 to 0.69, and less than 0.6 [11]. To exclude the possibility that the lower predictive performance of APACHE III may indeed stem, in part, from a beneficial effect of rosuvastatin, we performed sensitivity analysis by restricting to the control arm. Hosmer-Lemeshow goodness-of-fit statistic was used to assess the calibration of APACHE III in overall population and subgroups [12, 13]. The “pROC” package was used in R platform [14]. A non-significant P value was considered evidence of good calibration. Overall model performance was evaluated by using the Brier score. It measures the average squared deviation between predicted probabilities for a set of events and their outcomes. A lower score represents higher accuracy. The Brier score represents the overall performance of APACHE III in predicting outcome, involving both discrimination and calibration [15, 16].

The predictive value of APACHE III score might be moderate in ALI patients. To improve its predictive value we included other potential confounding variables that were found to be statistically significant in bivariate analysis into multivariable regression model. Furthermore, the primary cause of ALI was forced into the model because empirical evidence had suggested its association with mortality outcome in ALI patients [17].

Statistical analysis was performed by using STATA 13.1 (StataCorp, College Station, Texas 77845 USA) and R packages (version 3.1.1). Statistical significance was considered at p<0.05.

## Results

A total of 745 patients were included in the study, including 540 survivors and 205 non-survivors (Table 1). There was no statistical difference in gender, ethnicity, types of ICU, number of quadrants with infiltrates, and site of infection between survivors and non-survivors. Non-survivors were significantly older than survivors (59.71±16.17 vs 52±15.92 years, p<0.001). The primary causes of ALI were also different between survivors and non-survivors (p = 0.017). Survivors were more likely to have the cause of sepsis than non-survivors (21.2% vs. 15.1%). As expected, APACHE III score was higher in non-survivors than in survivors (106.72±27.30 vs. 88.42±26.86; p<0.001).

**Table 1:** Comparisons of baseline characteristics between survivors and non-survivors.

	Survivors (n = 540)	Nonsurvivors (n = 205)	p
Gender (Male No. %)	260 (48.1%)	105 (51.2%)	0.454
Age (years)*	52.00±15.92	59.71±16.17	<0.001
APACHE III*	88.42±26.86	106.72±27.30	<0.001
Ethnic			
Hispanic or Latino (No. %)	66 (12.2%)	20 (9.8%)	0.347
Race (No. %)			
White	434 (80.4%)	156 (76.1%)	0.199
Black or African American	75 (14.9%)	30 (14.6%)	0.794
Not reported	16 (3.0%)	8 (3.9%)	0.517
Location (No. %)			0.618
MICU	337 (62.4%)	131 (63.9%)	
SICU	27 (5.0%)	6 (2.9%)	
Cardiac SICU	3 (0.6%)	2 (1.0%)	
CCU	5 (0.9%)	2 (1.0%)	
Neuro ICU	15 (2.8%)	2 (1.0%)	
Burn	6 (1.1%)	3 (1.5%)	
Trauma	16 (3.0%)	3 (1.5%)	
MICU/SICU	126 (23.3%)	53 (25.9%)	
Others	5 (0.9%)	3 (1.5%)	
The number of quadrants with infiltrates (No. %)			0.292
2	94 (17.5%)	33 (16.1%)	
3	141 (26.2%)	44 (21.5%)	
4	303 (56.3%)	128 (62.4%)	
Infection site (No. %)			0.818
Thorax	386 (71.5%)	147 (71.7%)	
Abdomen	47 (8.7%)	18 (8.8%)	
Skin or soft tissue	24 (4.4%)	5 (2.4%)	
Bacterial meningitis	2 (0.4%)	2 (1.0%)	
Urinary tract	38 (7.0%)	13 (6.3%)	
Central line	1 (0.2%)	1 (0.5%)	
Osteomyelitis	2 (0.4%)	2 (1.0%)	
Confirmed Swine Influenza A	1 (0.2%)	0 (0.0%)	
Others	38 (7.0%)	16 (7.8%)	
Suspected infection^**$**^	1 (0.2%)	1 (0.5%)	
Primary causes of lung injury (No. %)^**¶**^			0.017
Trauma	6 (1.1%)	0 (0.0%)	
Sepsis	114 (21.2%)	31 (15.1%)	
Multiple transfusion	3 (0.6%)	1 (0.5%)	
Aspiration	41 (7.6%)	8 (3.9%)	
Pneumonia	370 (68.6%)	159 (77.6%)	
Other	5 (0.9%)	6 (2.9%)	
Fluid balance 24 hours preceding randomization (ml)	2083±2873	2423±2927	0.156
Use of vasopressor or inotrope (No. %)	278 (51.5%)	130 (63.4%)	0.003
CVP (mmHg)	11.8±4.8	11.6±5.0	0.633
Tidal volume (ml) ^**§**^	414±84	413±94	0.969
Weight (kg)	87.5±29.1	87.8±33.4	0.925
Creatinine (mg/dl)	1.47±1.16	1.69±1.24	0.033
Oxygenation index	167.0±70.6	158.7±61.5	0.117
Baseline MAP (mmHg)	77.5±13.4	74.8±14.3	0.03

Note:

* p<0.001 compared between survivors and nonsurvivors.

^**¶**^ The “primary” should be the most immediate cause. For example, a patient with multiple trauma who develops sepsis and then ALI: primary cause = sepsis; secondary cause = trauma.

^**§**^ For volume targeted mode.

^**$**^ Sepsis can be defined as proven or suspected. Suspected infection here means those without proven infection and the infection site is not known.

Abbreviations:

APACHE III: Acute Physiology and Chronic Health Evaluation III

ALI: acute lung injury.

MICU: medical ICU

SICU: surgical ICU

CCU: coronary care unit

MAP: mean arterial pressure.

**Fig 1:**
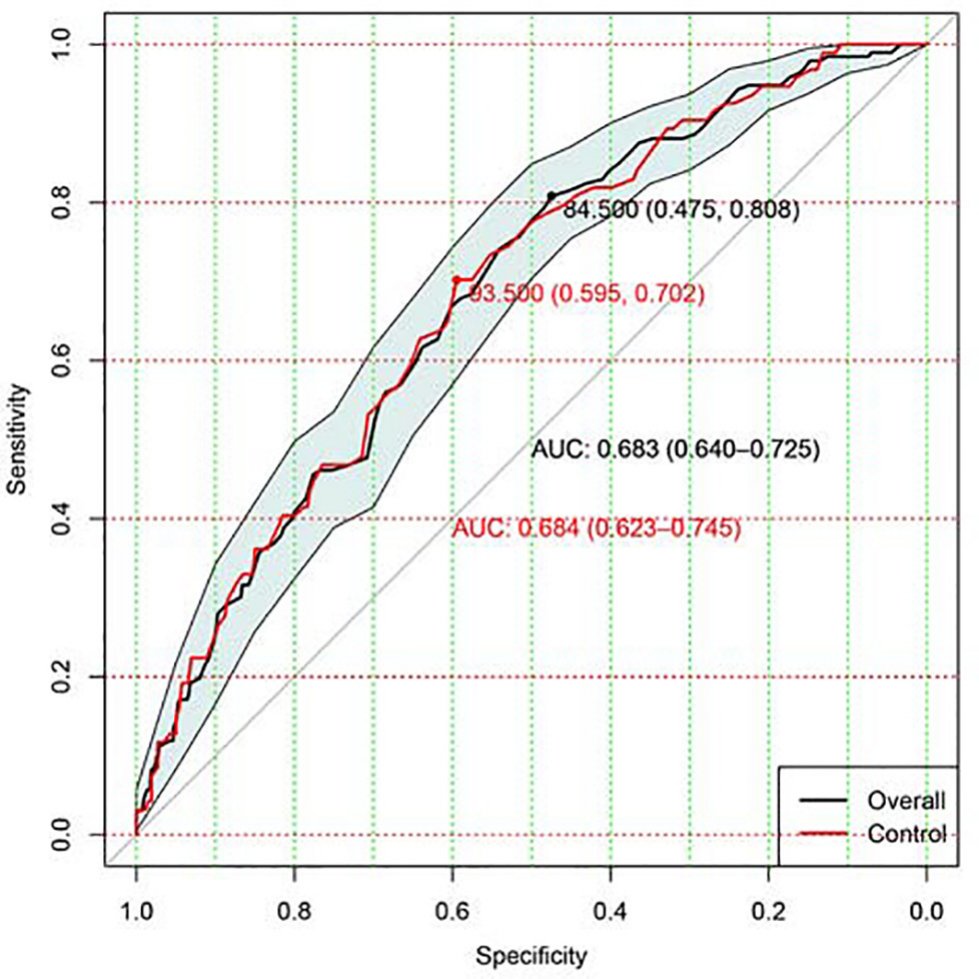
Assessment of discrimination with area under the receiver operating characteristic (ROC) curve. Discrimination of APACHE III to predict mortality in overall ALI patients was moderate with an AUC of 0.68 (95% CI: 0.64–0.73). The red line shows sensitivity analysis by restricting to patients in the control arm. The results showed an AUC of 0.68 (95% CI: 0.62–0.75).

**Fig 2:**
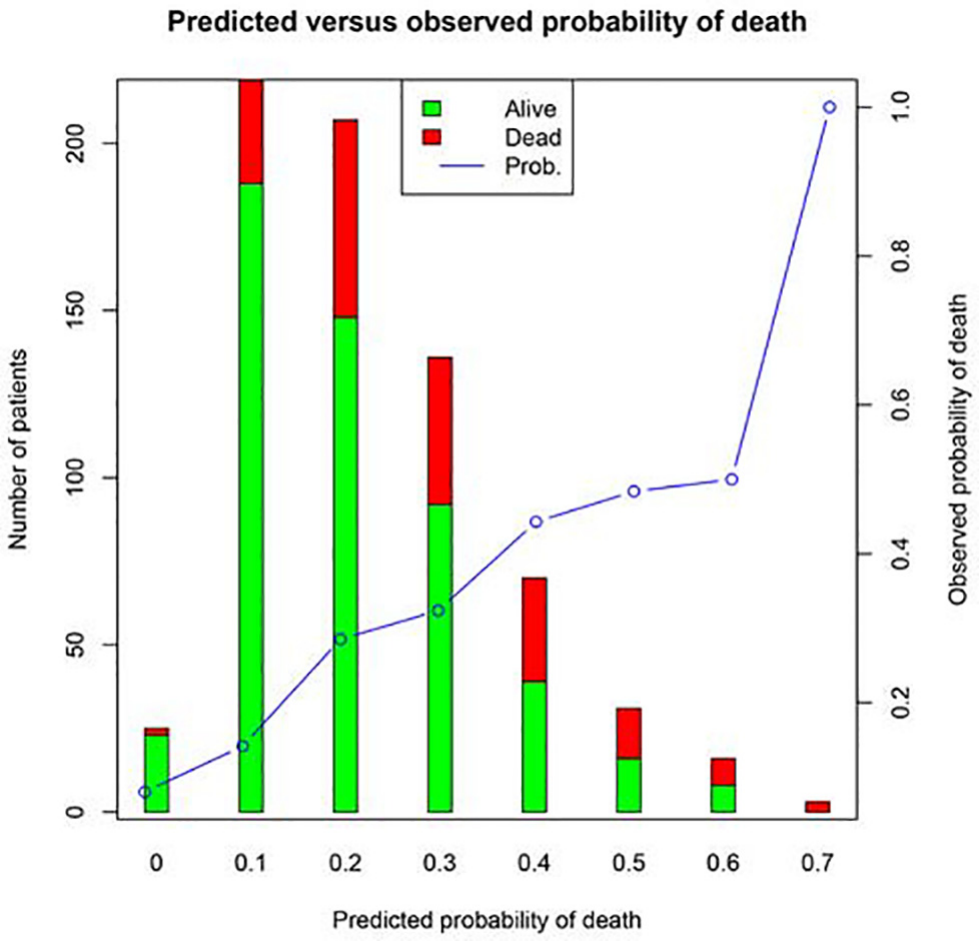
Predicted versus observed probability of death. The observed probability was calculated by categorizing predicted probability of death into eight subgroups. It is obvious that the observed probability of death increases monotonically.

Discrimination of APACHE III to predict mortality in ALI patients was moderate with an AUC of 0.68 (95% CI: 0.64–0.73; Fig 1). The model calibration was good with a non-significant p value for Hosmer-Lemeshow statistic (p = 0.61). Fig 2 shows calibration and discriminating of the APACHE III score in predicting mortality. Sensitivity analysis restricting to patients in control arm (n = 366) showed similar result with an AUC of 0.68 (95% CI: 0.62–0.75; red line in Fig 1). The Brier score was 0.1824, indicating that APACHE III had moderate predictive performance in ALI patients. The Hosmer-Lemeshow statistic was 6.31 (p = 0.61).

To adjust for confounding factors, we built a multivariable logistic regression model. The model initially incorporated all variables as listed in Table 1. Variables already included in calculating APACHE III score were omitted. However, all of the factors except for APACHE III were not statistically significant even at p = 0.1. Variable selection was performed by using stepwise procedure and only APACHE III was remained in the model. Thus, the result was actually univariate analysis as had been presented previously.

## Discussion

Although two studies have reported the discrimination of APACHE III in patients with ALI/ARDS, our study for the first time focused on patients with sepsis-associated ALI [18, 19]. The prior validations of APACHE III score included ARDS patients with heterogeneous etiologies and our study is novel in that ALI population was more homogenous. The result showed that the APACHE III predicts moderately for mortality outcome in ALI patients.

As the APACHE III was originally developed to predict in-hospital death [1], censored 60 days after enrollment for follow-up in our study deviates from the initial prognostic methodology, which may underlie the discrepancy between the original AUC and those presented within the current trial. The original APACHE III was developed almost 25 years ago. Thus, the advancements in critical care over this extended period (e.g. early goal directed therapy, low tidal volume ventilation and bundled care) and general reduction in overall ICU death many be another contribution to the discrepancy. It might also be worth noting that although the APACHE III score was developed in an “unselected” group of critically ill patients, its intended use was via the “APACHE III first-day hospital risk equation” in which a patient’s diagnosis (example “non-cardiogenic pulmonary edema”) modified the risk of death predicted (acknowledging that the score by itself would perform differently in patients with ARDS than unselected ICU patients).

Although it is still controversial, clinical outcomes of ALI may differ between pulmonary and extrapulmonary causes. Sepsis causes of ALI is a typical form of extrapulmonary cause, while aspiration and/or pneumonia are regarded as direct causes. Some studies reported adverse impact of sepsis, while others showed neutral effect [20–22]. In a cohort of ALI patients in MICU, Zilberberg MD and colleagues reported that sepsis was associated with increased risk of death. In our study, there was no evidence that sepsis was associated with increased risk of death (p>0.05). On the other hand, the definition of primary cause of ALI was different in our study. All patients had sepsis-associated ALI, but there were different priamry causes. For example, a patient with sepsis presents no sign of ALI at the very beginning. Then he or she may have aspiration because of vomiting during ICU stay and subsequently develops ALI. At this stage, he has sepsis-associated ALI, but the primary cause is aspiration. This distinct definition in our study may partly explain the conflicting results. Age was demonstrated to be an important risk factor for mortality, which is consistent with other reports [23, 24]. However, older patients generally have more medical comorbidities that contribute to their poor outcome.

The advantage of the study is the use of sepsis associated ALI subjects, enabling the minimization of clinical heterogeneity. The problem of heterogeneity is a critical issue in clinical researches involving ICU patients [25]. For example, the idiosyncrasy of one dataset collected from an ICU may cause a model developed with current dataset to be poorly validated in dataset collected in another ICU. There are substantial differences among ICUs from different hospitals in the type of patients, treatment protocols, and some other administration-related factors. By restricting to study population to sepsis associated ALI, we can minimize the problem of heterogeneity as much as possible. Secondly, the dataset we used was prospectively collected, which was more accurate and reliable than those collected retrospectively. For example, to determine the primary causes of ALI, we need prospective clinical observation that cannot be obtained with retrospective dataset.

Limitations of the study need to be acknowledged in the study. The treatment strategy and resuscitation protocol for those with sepsis were not incorporated in our analysis. Although these aspects have significant impact on clinical outcomes, they are not timely. The primary aim of prediction model is to provide prognostic information as early as possible. If we consider treatment protocols, the timeliness of the model will be compromised.

## Conclusion

In conclusion, the study for the first time validated the discrimination of APACHE III in sepsis associated ALI patients. The result showed that the APACHE III predicted moderately for mortality outcome in ALI patients.

### Key messages

- APACHE III predicted moderately for mortality outcome in ALI patients.
- The advantage of the study is the use of sepsis associated ALI subjects, enabling the minimization of clinical heterogeneity.

## Abbreviations

ICU: Intensive care unit
APACHE: Acute Physiology and Chronic Health Evaluation
SOFA: sequential organ failure score
ALI: acute lung injury
ROC: receiver operating characteristic
NHLBI: National heart, lung, blood institute
